# Invariant Recognition of Visual Objects: Some Emerging Computational Principles

**DOI:** 10.3389/fncom.2012.00060

**Published:** 2012-08-24

**Authors:** Evgeniy Bart, Jay Hegdé

**Affiliations:** ^1^Palo Alto Research CenterPalo Alto, CA, USA; ^2^Department of Ophthalmology, Vision Discovery Institute, and Brain and Behavior Discovery Institute, Georgia Health Sciences UniversityAugusta, GA, USA

Invariant object recognition refers to recognizing an object regardless of irrelevant image variations, such as variations in viewpoint, lighting, retinal size, background, etc. The perceptual result of invariance, where the perception of a given object property is unaffected by irrelevant image variations, is often referred to as perceptual constancy (Kofka, 1935; Walsh and Kulikowski, 2010).

Mechanisms of invariant object recognition have, to a significant extent, remained unclear. This is both because experimental and computational studies have so far largely focused on understanding object recognition without these variations, and because the underlying computational problems are profoundly difficult.

The 10 articles in this Research Topic Issue focus on some of the key computational issues in invariant object recognition. There is no pretending that the articles cover all key areas of current research exhaustively or seamlessly. For instance, none of the articles in this issue address size invariance (Kilpatrick and Ittelson, 1953) or color constancy (Foster, 2011). Nonetheless, the articles collectively paint a useful pointillist picture of current research on computational principles of invariance.

## Strategies of Representing Invariance

Several articles address various strategies of exploiting or representing the information in the visual image to achieve object invariance. Chuang et al. (2012) show, using psychophysical experiments, that non-rigid motion provides a cue to the invariance of dynamic objects. Groen et al. (2012) show that low-level image statistics can cue the extent to which natural textures are invariant across samples. Using electroencephalography (EEG), they also show that the differences in edge statistics predict the differences in the evoked neural responses to individual images. Using psychophysical experiments, Bart and Hegdé (2012)^1^ show that human subjects can use small informative fragments of an image to recognize an object regardless of variations in illumination. A more radical idea is proposed by Edelman and Shahbazi (2012), who argue that representing objects by their similarity to a set of prototypes can explain many properties of the visual system, including invariance.

## Strategies of Learning Invariance

In a supervised setting, cues to object invariance may be provided externally (e.g., Bart and Hegdé, 2012). In unsupervised settings, finding cues to invariance is more challenging. One type of cues arises from the fact that even when an object changes in appearance, the change is generally smooth. Thus, over short, selected stretches of space and/or time, the changes in object appearance tend to be rather small, so that the visual system can, in principle, infer that the same object is changing its appearance. A theoretical approach for exploiting this contiguity is given by the continuous transformation (CT) learning (Stringer et al., 2006). A related cue arises from the fact that objects often stay in view for extended periods of time; two observations at nearby time points are therefore likely to correspond to the same object. An approach that exploits this temporal contiguity is given by the trace learning rule (Földiák, 1991).

Many articles in this issue describe models that exploit one or more of these rules to learn object invariance. The VisNet model can incorporate one or both of these strategies, depending on the particular implementation. The article by Rolls (2012) describes the various capabilities of VisNet. The article by Tromans et al. (2012) highlights the capability of VisNet to learn with clutter and occlusion. VisNet, like most neural network models, uses rate coding, in which the firing rate of a neuron determines the information coded by that neuron. The firing rate of a neuron is usually specified as a scalar, without the neuron having to actually fire spikes. The article by Evans and Stringer (2012) implements VisNet in which individual neurons actually fire spikes, and detail the merits of this implementation. The model by Isik et al. (2012) describes a different model, HMAX (also see Serre et al., 2007), that simulates many invariance properties in the primate visual system.

It is worth noting that, while it is generally thought that object invariance is represented by neurons in the higher levels of the visual pathway, such as the inferotemporal cortex, neurons in the lower levels, such as the primary visual cortex or V1, can also play key roles in implementing various aspects of invariance. The article by Vidal-Naquet and Gepshtein (2012) shows that populations of V1 complex cells, but not individual complex cells, can compute information about stereoscopic disparity in a spatially invariant fashion.

## Some Important Caveats

It is important to emphasize a few caveats about the implications of these articles for future research. First, at the perceptual level, object invariance neither is perfect nor needs to be (Bülthoff and Edelman, 1992; DiCarlo and Cox, 2007). Thus, the underlying neural mechanisms need not deliver perfect invariance. Second, not all types of invariance are equal. Some types of invariance may be more important or useful to the visual system than others, depending on the behavioral context (see Milivojevic, 2012). Third, the visual system does not necessarily have to rely on prolonged supervised learning to learn invariance. It is possible that the system is able to either learn or, alternatively, infer invariance on the fly, and without any feedback (see Rolls, 2012). Fourth, top-down factors, such as the behavioral context, play an important role in object invariance and lack thereof. This is not fully addressed by the articles in this issue, which mostly focus on bottom-up processing of invariance information. Finally, for practical reasons, current research tends to deal with invariance along the various individual stimulus parameters (e.g., viewpoint, illumination, etc.) separately from each other. But in actuality, the visual system may combine invariance across multiple visual parameters, and indeed multiple sensory modalities.

## Footnotes

^1^Who are also the editors of this Research Topic Issue and the authors of this editorial.
